# Mitochondrial complex I derived ROS regulate stress adaptation in *Drosophila melanogaster*

**DOI:** 10.1016/j.redox.2020.101450

**Published:** 2020-02-07

**Authors:** Filippo Scialò, Ashwin Sriram, Rhoda Stefanatos, Ruth V. Spriggs, Samantha H.Y. Loh, L. Miguel Martins, Alberto Sanz

**Affiliations:** a Institute for Cell and Molecular Biosciences, Newcastle University Institute for Ageing, Newcastle University, Campus for Ageing and Vitality, Newcastle upon Tyne, NE4 5PL, United Kingdom; b MRC Toxicology Unit, University of Cambridge, Hodgkin Building, Lancaster Road, Leicester, LE1 9HN, United Kingdom; c Institute of Molecular, Cell and Systems Biology, College of Medical, Veterinary and Life Sciences, University of Glasgow, G12 8QQ, Glasgow, United Kingdom

**Keywords:** Heat stress, Reverse electron transport, Complex I, Reactive oxygen species, Alternative oxidase, AOX

## Abstract

Reactive Oxygen Species (ROS) are essential cellular messengers required for cellular homeostasis and regulate the lifespan of several animal species. The main site of ROS production is the mitochondrion, and within it, respiratory complex I (CI) is the main ROS generator. ROS produced by CI trigger several physiological responses that are essential for the survival of neurons, cardiomyocytes and macrophages. Here, we show that CI produces ROS when electrons flow in either the forward (Forward Electron Transport, FET) or reverse direction (Reverse Electron Transport, RET). We demonstrate that ROS production via RET (ROS-RET) is activated under thermal stress conditions and that interruption of ROS-RET production, through ectopic expression of the alternative oxidase AOX, attenuates the activation of pro-survival pathways in response to stress. Accordingly, we find that both suppressing ROS-RET signalling or decreasing levels of mitochondrial H_2_O_2_ by overexpressing mitochondrial catalase (mtCAT), reduces survival dramatically in flies under stress. Our results uncover a specific ROS signalling pathway where hydrogen peroxide (H_2_O_2_) generated by CI via RET is required to activate adaptive mechanisms, maximising survival under stress conditions.

## Highlights

• Heat stress induces ROS generation through the activation of Reverse electron transport (ROS-RET).
• Suppressing ROS-RET attenuates the transcriptional stress response reducing survival under stress.
• Mitochondrial catalase reduces survival under stress, identifying H_2_O_2_ as the main ROS signalling molecule.

## Graphical abstract

**Figure:**
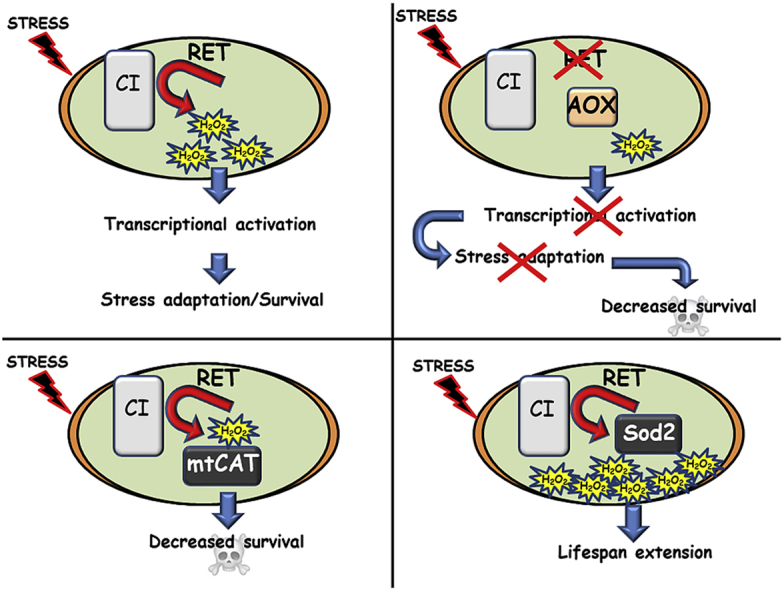

## Abbreviations

ROS: reactive oxygen species
CI: complex I
CIII: complex III
CoQ: coenzyme-Q
RET: reverse electron transport
FET: forward electron transport
NDI1: NADH dehydrogenase internal 1
AOX: alternative oxidase
ETC: electron transport chain
mtCAT: mitochondrial catalase
SOD2: superoxide dismutase 2
H_2_O_2_: hydrogen peroxide
FCCP: carbonyl cyanide-4-(trifluoromethoxy) phenylhydrazone
ROT: rotenone
Δp: proton motive force
ATP: adenosine triphosphate

## 1 Introduction

ROS are intriguing molecules. When animals age, they accumulate damaged mitochondria that produce high levels of ROS [1]. However, clinical trials have shown that in the majority of cases administering antioxidants is not beneficial [2]. In fact, increasing mitochondrial ROS levels has been shown to extend lifespan in several animal species [[3], [4], [5], [6]]. These contradictions serve to emphasise the dual nature of ROS. They are metabolic by-products that can cause oxidative damage [7], but ROS are also important messengers required for cellular homeostasis [8]. The amount of ROS is important and may explain many of the contradictory effects of free radicals [9], however the time, location and nature of the ROS generated are also key in determining their physiological effects [10]. Only by understanding the relationship between these factors we will be able to develop effective interventions to promote the positive effects of ROS, while reducing the negative.

Mitochondrial CI is the main source of ROS [11] and manipulation of its activity, alters animal lifespan [4,12,13]. Within CI electrons can flow in either the forward or reverse direction. Normally, electrons flow in the forward direction, from CI to CIII via Coenzyme-Q (CoQ). However, electrons can, in certain conditions, also flow back from ubiquinol (reduced CoQ or CoQH_2_) to CI, generating a significant amount of ROS. This process is known as RET and occurs in several animal species in physiological and pathological conditions [14]. ROS-RET depends both on the proton motive force (Δp) and redox state of the CoQ pool [15], which are linked to ATP generation and electron flow respectively. Δp determines how much ATP the mitochondrion can produce, while CoQ acts as crossroads where several metabolic pathways, including glycolysis, Krebs cycle, fatty acid oxidation and pyrimidine biosynthesis, meet. Therefore, coupling RET to ROS production is a very efficient way to communicate information from the mitochondrion to other parts of the cell. In fact, ROS-RET signalling is known to trigger cardiorespiratory adaptations in response to changes in oxygen levels [16], regulate sleep patterns in flies [17], reprogram macrophage metabolism in response to bacterial infection [18], alleviate impairment caused by interruption of electron flow [19] and suppress cell death and tissue damage in episodes of ischemia-reperfusion [20].

Considering how implicated CI is in human ageing and age-related diseases, it is important to understand how ROS-RET signalling operates *in vivo* and if it has a role in the determination of longevity. We have previously shown that inducing ROS-RET through the expression of the NADH dehydrogenase internal 1 (NDI1) preserves mitochondrial function and extends lifespan in *Drosophila melanogaster* [13]. Here, we extend this work demonstrating that ROS-RET signalling occurs physiologically in the brain of wild type *Drosophila melanogaster* flies in response to heat stress. Furthermore, to study the role of ROS-RET in stress adaptation, we took advantage of alternative oxidase (AOX), which is not present in the electron transport chain (ETC) of humans or fruit flies, but is expressed in plants, fungi and many animal species [21]. AOX reduces the generation of ROS by preventing the over-reduction of the ubiquinone pool [22,23]. Here we demonstrate that ectopic expression of AOX prevents the activation of ROS-RET resulting in the downregulation of a pro-survival transcriptional response that in turn causes a negative effect on the survival of flies under different types of stress. We show that the ectopic expression of mtCAT within the mitochondrial matrix phenocopies the effects of AOX expression, whereas the overexpression of Superoxide dismutase (Sod2) has a positive effect on longevity. We extend the significance of our discoveries showing that adaptation to other stresses such as different levels of oxygen also requires a mitochondrial H_2_O_2_ signal. Finally, we dissect the nature of this ROS-RET signal and identify and implicate mitochondrial H_2_O_2_ in lifespan regulation. Our results validate the manipulation of ROS produced by CI *in vivo* as a strategy to maximise survival under stress conditions and advise against implementing antioxidant strategies that completely suppress mitochondrial H_2_O_2_ signalling.

## 2 Material and methods

### 2.1 Fly stocks and lifespan experiments

All UAS transgenes and GAL4 driver lines were backcrossed for at least six generations into our *white* Dahomey (wDAH) background [24] unless otherwise stated. UAS-AOX flies have previously been described in Ref. [25]. UAS-mito-Catalase (mtCAT) flies were a kind gift from Professor Rajindar Sohal (Bayne et al., 2005), UAS-Sod2 and daughterless-GAL4 (daGAL4) were obtained from the Bloomington Drosophila Stock Center (BDSC). The RNAi line against ND-75 (100733/KK) and the control w^1118^ strain were obtained from the Vienna Drosophila Resource Center (VDRC) and were used without backcrossing into wDAH.

**Fig. 1:**
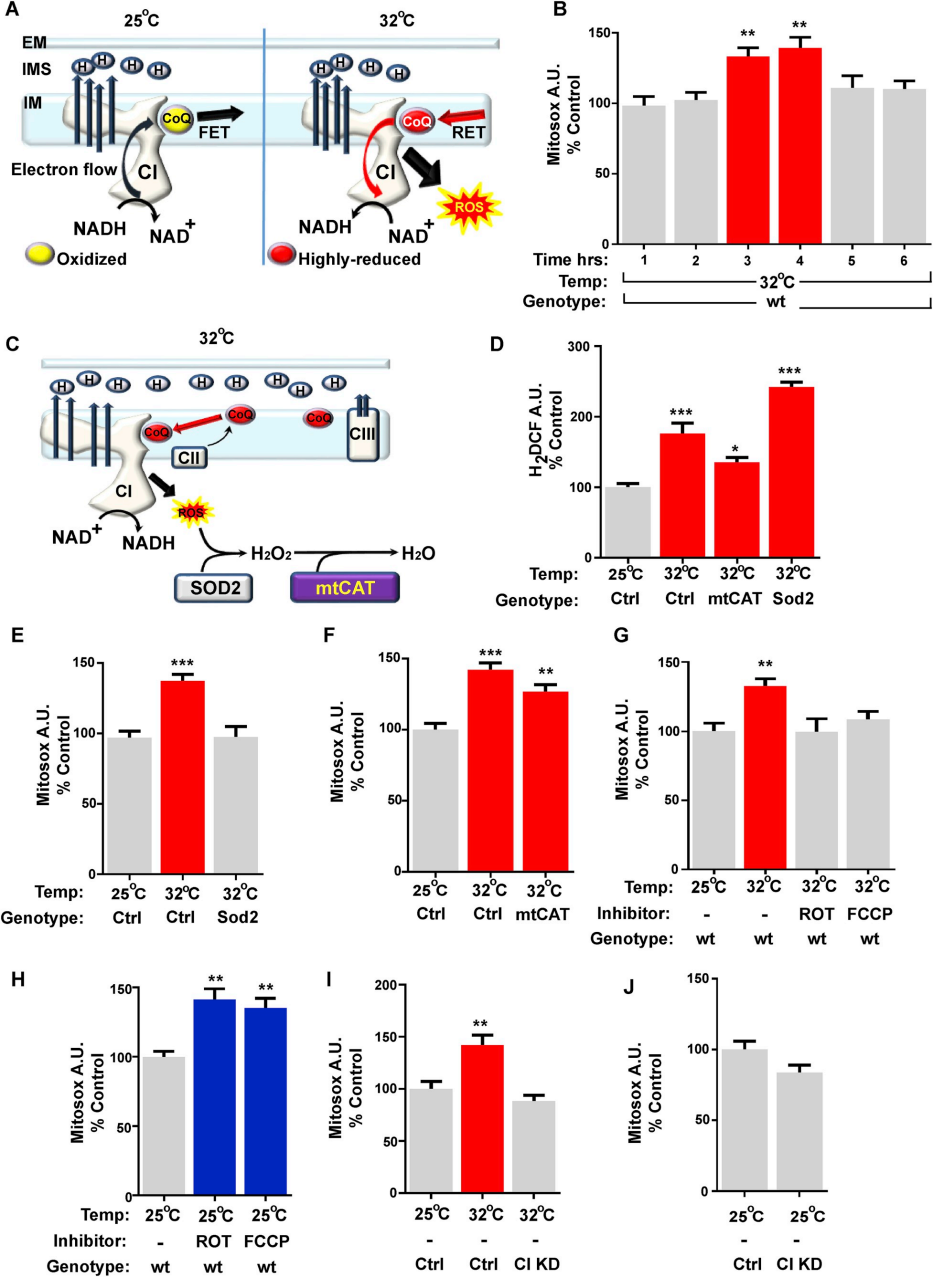
Heat stress induces ROS production through the activation of Reverse Electron Transport. **A)** Schematic representation of electron flow through CI during Forward Electron Transport (FET) at 25^o^C and Reverse Electron Transport (RET) at 32^o^C. **B)** Time course analysis of mitochondrial ROS (MitoSOX quantification) in fly brains from flies exposed to heat stress (32^o^C). Measurements were taken at six time points. Results are expressed in relation to control flies maintained at 25^o^C. **C)** Schematic representation of how Sod2 and mtCAT modulate ROS levels in the mitochondrial matrix. **D)** Analysis of mitochondrial ROS (H_2_DCF) in fly brains from controls (Ctrl = 2>daGAL4, 25 °C), control flies exposed to heat stress (32^o^C, 3 h) (2>daGAL4) or flies overexpressing mtCAT (mtCAT > daGAL4) or Sod2 (Sod2>daGAL4) exposed to heat stress. **E)** Analysis of mitochondrial ROS (MitoSOX) in fly brains from controls (Ctrl = 2>daGAL4, 25^o^C) or control flies exposed to heat shock (32^o^C, 3 h) (2>daGAL4) or flies overexpressing Sod2 (Sod2>daGAL4). **F)** Analysis of mitochondrial ROS (MitoSOX) in fly brains from controls (Ctrl = 2>daGAL4, 25^o^C), control flies exposed to heat stress (32^o^C, 3 h) (2>daGAL4) or flies overexpressing mtCAT (mtCAT > daGAL4). **G)** Analysis of mitochondrial ROS (MitoSOX) in brains from controls (25^o^C) or flies exposed to heat stress (32^o^C, 3 h) in the presence or absence of the indicated mitochondrial inhibitor. **H)** Analysis of mitochondrial ROS (MitoSOX) in brains from controls (25^o^C) flies exposed in the presence or absence of the indicated mitochondrial inhibitor during 3 h. **I)** Analysis of mitochondrial ROS (MitoSOX) in brains from control (Ctrl = ND75-IR>3, at 25^o^C) or flies exposed to heat stress (32^o^C, 3 h) with normal (Ctrl = ND75-IR>3) or reduced levels of respiratory CI (CI KD = ND75-IR > daGAL4). **J)** Analysis of mitochondrial ROS (MitoSOX) in fly brains from controls (Ctrl = ND75-IR>3) and flies with reduced levels of respiratory CI (CI KD = ND75-IR > daGAL4). Values shown represent means ± SEM of between 6 and 9 biological replicates. *p < 0.05, **p < 0.01, ***p < 0.001.

**Fig. 2:**
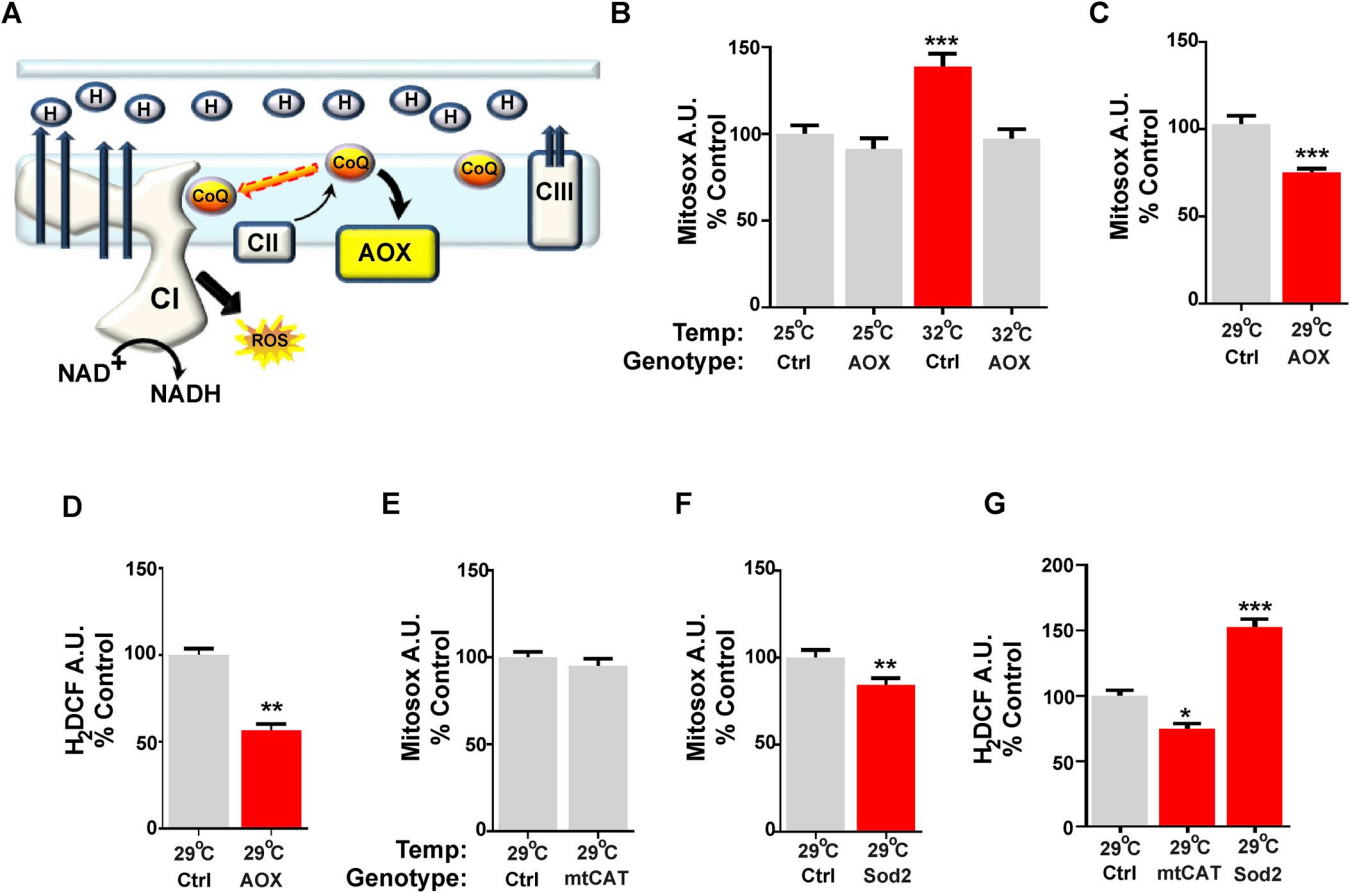
AOX inhibits the generation of ROS-RET. **A)** Schematic representation of how AOX prevents production of ROS-RET. **B)** Analysis of mitochondrial ROS (MitoSOX) in fly brains from control (Ctrl = 2>daGAL4) and AOX (AOX > daGAL4) flies cultured at 25^o^C or exposed to heat stress (32 °C, 3 h). **C)** Analysis of mitochondrial ROS (MitoSOX) in fly brains from control (Ctrl = AOX>3) and AOX flies (AOX > daGAL4) cultured at 29^o^C for 10 days. **D)** Analysis of mitochondrial ROS (H_2_DCF) in fly brains from control (Ctrl = 2>daGAL4) and AOX (AOX > daGAL4) flies cultured at 29^o^C for 10 days. **E)** Analysis of mitochondrial ROS (MitoSOX) in fly brains from control (Ctrl = 2>daGAL4) and mtCAT flies (mtCAT > daGAL4) cultured at 29^o^C for 10 days. **F)** Analysis of mitochondrial ROS (MitoSOX) in fly brains from controls (Sod2>3) and Sod2 flies (Sod2>daGAL4) cultured at 29 °C for 10 days. **G)** Analysis of mitochondrial ROS (H_2_DCF) in fly brains from control (Ctrl = 2>daGAL4), mtCAT (mtCAT > daGAL4) and Sod2 flies (Sod2>daGAL4) cultured at 29 °C for 10 days. Values shown represent means ± SEM of between 9 biological replicates. *p < 0.05, **p < 0.01, ***p < 0.001.

**Fig. 3:**
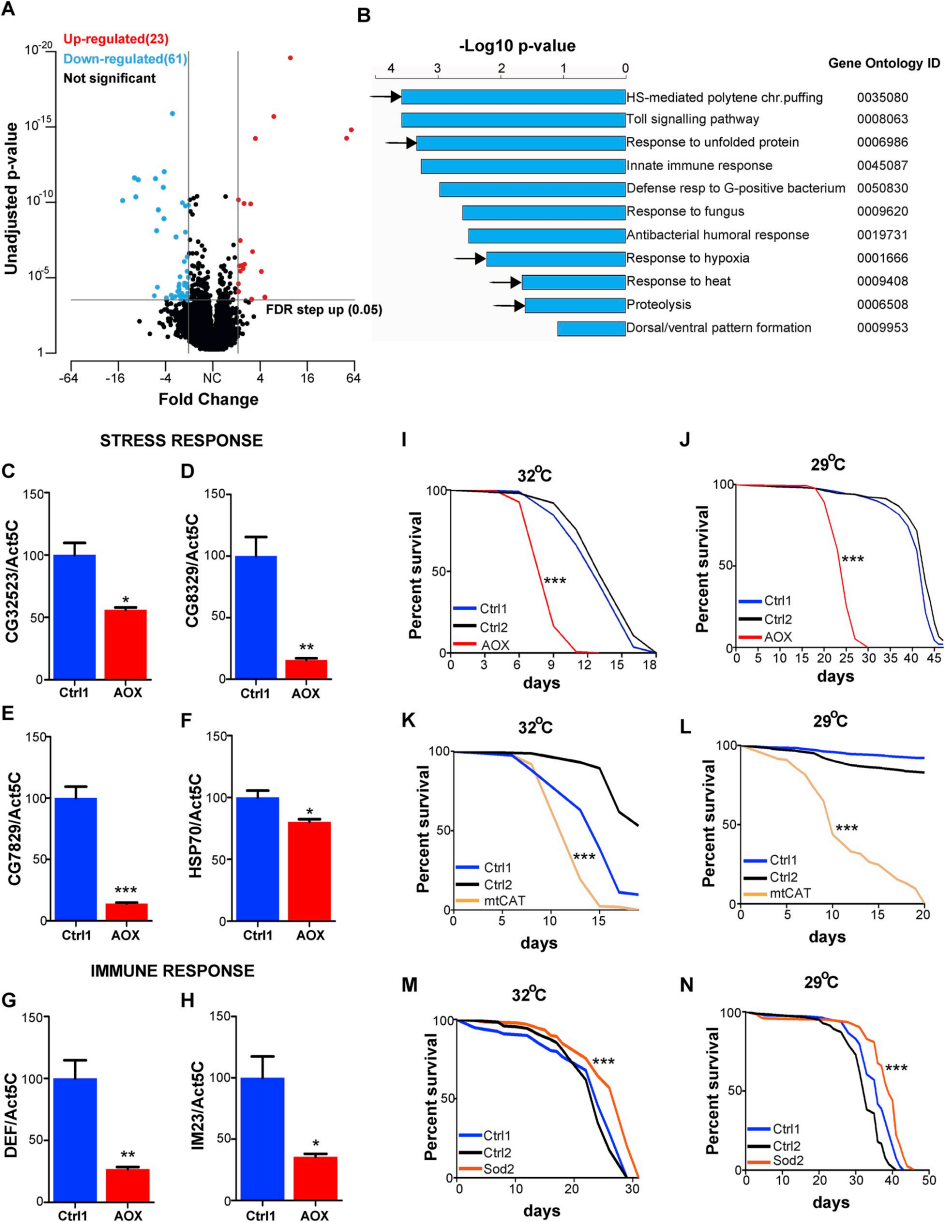
Abolishing ROS-RET diminishes the transcriptional stress response and shortens survival under stress. **A)** Volcano Plot showing transcripts up- (red) or down-regulated (blue) by AOX expression. **B)** GO Analysis showing biological pathways enriched with genes downregulated by AOX. Arrows point to pathways belonging to stress response. **C)***CG3*2523 mRNA level in heads of control (Ctrl1 = 2>daGAL4) and AOX flies (AOX > daGAL4) normalised to *Act5C* mRNA. Three biological replicates per group. Student t-test. Error bars represent standard error of the mean (SEM). * denotes p < 0.05. **D)***CG8*329 mRNA level in heads of control (Ctrl1 = 2>daGAL4) and AOX flies (AOX > daGAL4) normalised to *Act5C* mRNA. Three biological replicates per group. Student t-test. Error bars represent standard error of the mean (SEM). ** denotes p < 0.01. **E)***CG7*829 mRNA level in heads of control (Ctrl1 = 2>daGAL4) and AOX flies (AOX > daGAL4) normalised to *Act5C* mRNA. Three biological replicates per group. Student t-test. Error bars represent standard error of the mean (SEM). *** denotes p < 0.001. **F)***Hsp7*0 mRNA level in heads of control (Ctrl1 = 2>daGAL4) and AOX flies (AOX > daGAL4) normalised to *Act5C* mRNA. Three biological replicates per group. Student t-test. Error bars represent standard error of the mean (SEM). **G)***Def* mRNA levels in heads of control (Ctrl1 = 2>daGAL4) and AOX flies (AOX > daGAL4) normalised to *Act5C* mRNA. Three biological replicates per group. Student t-test. Error bars represent standard error of the mean (SEM). ** denotes p < 0.01. **H)** Im*2*3 mRNA level in heads of control (Ctrl1 = 2>daGAL4) and AOX flies (AOX > daGAL4) normalised to *Act5C* mRNA. Three biological replicates per group. Student t-test. Error bars represent standard error of the mean (SEM). * denotes p < 0.05. **I)** Survival of control (Ctrl1 = 2>daGAL4 and Ctrl2 = AOX>3) and AOX flies (AOX > daGAL4) cultured at 32^o^C. **J)** Survival of control (Ctrl1 = 2>daGAL4 and Ctrl2 = AOX>3) and AOX flies (AOX > daGAL4) cultured at 29^o^C. **K)** Survival of control (Ctrl1 = mtCAT>3 and Ctrl2 = 2>daGAL4) and mtCAT flies (mtCAT > daGAL4) cultured at 32^o^C. **L)** Survival of control (Ctrl1 = mtCAT>3 and Ctrl2 = 2>daGAL4) and mtCAT flies (mtCAT > daGAL4) cultured at 29^o^C. **M)** Survival of control (Ctrl1 = 2>daGAL4 and Ctrl2 = Sod2>3) and Sod2 flies (Sod2>daGAL4) cultured at 32^o^C. **N)** Survival of control (Ctrl1 = 2>daGAL4 and Ctrl2 = Sod2>3) and Sod2 flies (Sod2>daGAL4) cultured at 29^o^C. Lifespan data were analysed using the log-rank Mantel Cox Test. *p < 0.05, **p < 0.01, ***p < 0.001.

Flies were maintained on standard media (1% agar, 1.5% sucrose, 3% glucose, 3.5% dried yeast, 1.5% maize, 1% wheat, 1% soya, 3% treacle, 0.5% propionic acid, 0.1% Nipagin), collected using CO_2_ anaesthesia within 24 h of eclosion and then maintained at a density of 20 flies per vial at the desired temperature (25 °C, 29 °C or 32 °C). Flies were transferred to fresh vials every 2–3 days. Lifespan experiments were performed with a minimum of 100 flies per genotype and repeated at least twice. For experiments performed in hypoxia and hyperoxia conditions, flies were cultured at 5% and 50% oxygen levels respectively at 25 °C and transferred to fresh vials once every seven days (hypoxia) or four days (hyperoxia) to avoid detrimental effects due to reoxygenation. The number of dead flies was recorded every 2–3 days, and the median lifespan was calculated for each experiment. Flies between 2 and 5 days (experiments in Fig. 1, Fig. 2B) or 10–15 days old (Fig. 2C–G and Fig. 3C–H) were used in all experiments unless otherwise stated. Inhibitors of ETC dissolved in ethanol were added to the fly food at a final concentration of 600 μM ROT (Sigma) and 600 μM FCCP (Sigma).

### 2.2 Measurement of ROS in *Drosophila* brains

MitoSOX and 2’,7’-dichlorofluorescein (H_2_DCF) were used to detect either mitochondrial matrix superoxide or total levels of peroxides, respectively. Brains were dissected in phosphate-buffered saline (PBS). Following dissection, brains were incubated in either 20 μM MitoSOX or 30 μM H_2_DCF for 10 min, washed three times with PBS 1X and imaged immediately. Images were acquired using a Leica SP8 confocal digital Light Sheet (Leica microsystem) or LSM510 confocal microscopes (Zeiss) both equipped with a 10x 0.3 NA objective as z stacks throughout the sample, using either a 543 nm HeNe laser or a 488 nm line of an Argon laser to excite MitoSOX and H_2_DCF respectively. The total average fluorescence intensity of each brain imaged was quantified using ImageJ.

### 2.3 Next-generation sequence data acquisition and analysis

RNA was extracted from fly heads (20 heads per sample, five replicates for each genotype or condition). Heads were homogenised in TRI Reagent (Sigma) by using a motorised pestle and following the manufacturer's instructions. RNA was treated with DNase I (Thermo Fisher Scientific) at 37 °C for 60 min and precipitated overnight with 3 M sodium acetate and 95% ethanol. After centrifugation, pellets were dissolved in an appropriate volume of DNase/RNase free water. The RNA quality was confirmed using an Agilent 2100 Bioanalyzer (Agilent Technologies, CA, USA). Detailed experimental protocols and raw data were deposited in ArrayExpress under accession E-MTAB-7952. Briefly, NGS data acquisition was performed using the TruSeq Stranded mRNA kit (Illumina) following the manufacturer's instructions. Raw data were acquired using an Illumina sequencer (NextSeq500) and processed using Partek Flow (Partek Inc. Missouri, USA). RNA reads were normalised using the default method (total count, add 0.0001) and aligned to Reference Index BDGP6 using STAR 2.4. d.

To select transcripts that were up- or down-regulated in AOX expressing flies for GO Analysis, we filtered transcripts whose Fold change (FC) expression was ±2, discarding those whose FDR was above 5%. These selected transcripts were analysed using DAVID [26]. The GOTERM_BP_DIRECT in the Gene Ontology section of the Annotation Summary Results was used to retrieve the list of GOTERM.

### 2.4 RNA extraction, cDNA synthesis and qPCR

RNA extraction was performed from fly heads as described previously. cDNA synthesis and qPCR was performed as described in Ref. [27]. Briefly, cDNA synthesis was achieved using the High-Capacity cDNA Reverse Transcription Kit (Fisher Scientific, Applied Biosystems™; 4368814). qPCR: qPCR was carried out using QuantiNova SYBR® Green PCR Kit (Cat.no. 208056, QIAGEN), and the following primers were used: *CG32523* (forward primer: AGTGAATCCGCGATAGAGCC, reverse primer: CCACGTAGACGCAGGGAAAT), *CG8329* (forward primer: CAATGGAGGATTGGCCGACT, reverse primer: CCACCGATCCGTATGACCTG), *CG7829* (forward primer: CATGAATGGTCCTCCCTCGG, reverse primer: CCGATCCGTCACAGTTTTGC), *hsp70* (forward primer:, reverse: primer), *def* (forward primer: AGCCACATGCGACCTACTCT, reverse primer: GTTGCAGTAGCCGCCTTTGA), *im23* (forward primer: GTGCCTGATTCTGTCCTTTGC, reverse primer: TGCAATCCTGGCATACTCCG). *act5c* was used as internal standard (forward primer: GAGGAAGCAGCAGCGAAAGT, reverse primer: TTTTGTTGTGCTGCACTCCAA).

### 2.5 Statistical analysis

The data were analysed using GraphPad Prism 6 software using either the unpaired Student's *t*-test or One-way ANOVA with Dunnett's post-test where appropriate unless otherwise stated. Lifespan data were analysed using the Kaplan Meier Log-Rank Test.

## 3 Results and discussion

### 3.1 Heat stress modifies CI ROS production

We have previously reported that induction of ROS-RET signalling through ectopic expression of yeast-derived NDI1 improved mitochondrial function and increased lifespan [13]. Here, we wanted to understand if ROS-RET signalling could be induced physiologically and if so, how and which role its activation plays in the determination of longevity. Since RET requires a highly reduced CoQ pool and heat increases mitochondrial oxygen consumption (data not shown), we decided to study whether heat stress modified the production of ROS from mitochondria. We observed that exposing flies to heat stress (32 °C) led to increased ROS in the fly brain after 3 h (Fig. 1A and B). To confirm that this increase in ROS was mitochondrial in origin, we overexpressed two different mitochondrial antioxidants, Sod2 and mtCAT, which detoxify superoxide and H_2_O_2_ respectively (Fig. 1C). We found that overexpression of either Sod2 or mtCAT attenuated the levels of ROS produced in response to heat stress (Fig. 1D and E) confirming that mitochondria are the primary source of ROS. We observed that Sod2 overexpression improved the detoxification of superoxide and in the process increased H_2_O_2_ (Fig. 1D and E). Conversely, mtCAT overexpression did not have any effect on superoxide level (Fig. 1F) but decreased the level of H_2_DCF under stress (Fig. 1D).

To test whether this ROS increase was as a result of RET, we fed flies with either rotenone (ROT) or carbonyl cyanide-4-(trifluoromethoxy) phenylhydrazone (FCCP). ROT is a CI inhibitor that binds to the quinone binding site preventing electrons from flowing backwards, whereas FCCP dissipates the Δp also required for RET [14,15]. Supporting our hypothesis, we found that both treatments were sufficient to prevent increased ROS production in response to heat stress (Fig. 1G). While, in flies not exposed to heat stress, feeding with either ROT or FCCP for 3 h at 25°C resulted in increased levels of ROS compared to vehicle fed controls (Fig. 1H), indicating FET through CI. Finally, to implicate CI as the source of ROS-RET, we expressed dsRNA against a catalytic subunit of CI: *ND-75* [4]. Knock-down of *ND-75* did not alter ROS levels at 25°C (Fig. 1J) but suppressed the RET derived ROS increase in response to heat stress (Fig. 1I). Together this data demonstrates that RET can be induced physiologically in the fly brain leading to ROS production via CI.

### 3.2 The alternative oxidase AOX inhibits the generation of the ROS-RET signal

To test the hypothesis that ROS-RET could have a leading role in triggering the response to stress adaptation and in doing so have a primary role in determination of longevity, we decided to study the effect of the long-term inhibition of this signal. To avoid unintended metabolic consequences of CI inhibition with ROT (e.g. acidosis), we chose to allotopically express the alternative oxidase (AOX) from *Ciona intestinalis* (Fig. 2A)*.* AOX prevents RET in cells, flies, and mice without interrupting electron flow [13,18,22]. Furthermore, in standard culture conditions (25 °C) AOX expression does not alter survival [28]. We confirmed that AOX abolishes ROS-RET signalling in flies exposed to heat stress and detected no significant changes to ROS levels in standard conditions (Fig. 2B). Since we were interested in the role of ROS-RET activation in longevity, we chose a moderate level of stress over severe, as the former has been used as a model for accelerated ageing [29] and flies are still able to mate and fertilised eggs develop normally at this temperature (29 °C). We confirmed that after 10 days of moderate heat stress ROS levels were decreased in AOX flies compared to controls (Fig. 2C and D). This indicates that AOX activity is maintained at this temperature, preventing the generation of ROS-RET. Furthermore, overexpression of Sod2 or mtCAT was able to attenuate the levels of ROS produced in response to long-term heat stress (Fig. 2F and G).

### 3.3 Suppression of the ROS-RET signal attenuates the transcriptional heat stress response essential for survival under stress

To further understand the long-term consequences of suppressing ROS-RET, we performed a transcriptomic analysis of brains from control and AOX expressing flies exposed to moderate heat stress (29 °C) for ten days. Using RNA sequencing analysis, we identified 84 different transcripts whose expression was significantly altered by AOX (Fig. 3A; Table 1). Gene ontology (GO) analysis showed that genes downregulated in AOX flies were included within GO terms associated with stress response (Fig. 3B black arrows). In addition, we observed a reduction in the expression of genes belonging to stress response pathways related to the immune response against both bacteria and fungi (GO0008063, GO0045087, GO050830, GO0009620, GO0019731). The reduction we observed in the pathogen response is not unexpected as ROS generally [30] and ROS-RET specifically [18] are instrumental in the activation of the immune response in mammals. We confirmed the RNAseq data selecting representative genes for the stress response pathway and immune response and checked their level by quantitative PCR (Fig. 3C–H). Next, we analysed the survival of AOX flies under severe (32 °C) and moderate (29 °C) heat stress. In line with the requirement of ROS-RET signalling for stress adaption, AOX expression dramatically shortened survival under severe and moderate heat stress (Fig. 3I and J). A similar situation is observed when AOX is expressed “to correct” the increase in mitochondrial ROS found in COX15 (a CIV subunit) knockout (KO) mice [19]. COX15 KO mice live much shorter than controls, and lifespan of these mice is further shortened when AOX is expressed. Besides reducing ROS levels, AOX also prevents the expression of many stress response genes in COX15 KO mice [19]. These results suggest that ROS-RET signalling is necessary for adaptation to several stressors in flies and mice and that this adaptation is at least partially coordinated at the transcriptomic level.

**Table 1:** **A)** List of genes identified by Gene Ontology Analysis shown in Fig. 3B.

**AOX up-regulated**							
**Gene_ID**	**Transcript_ID**	**Total reads**	**Q-value**	**P-value**	**FDR_step_up**	**Ratio**	**Fold_change**
			(daGal4>AOX_vs_daGal4)	(daGal4>AOX_vs_daGal4)	(daGal4>AOX_vs_daGal4)	(daGal4>AOX_vs_daGal4)	(daGal4>AOX_vs_daGal4)
FBgn0052865	FBtr0082678	295,68	1,64E-13	3,43E-17	2,33E-13	58,11	58,11
FBgn0050083	FBtr0300381	90,29	1,17E-12	3,26E-16	1,66E-12	50,59	50,59
FBgn0003996	FBtr0070490	405,09	1,69E-17	1,18E-21	2,41E-17	9,72	9,72
FBgn0013278	FBtr0082637	3612,73	2,79E-14	3,9E-18	3,97E-14	5,98	5,98
FBgn0000711	FBtr0071447	399,01	0,0131	0,000115	0,0186	4,59	4,59
FBgn0040384	FBtr0309881	58,98	0,000668	0,00000256	0,000949	4,15	4,15
FBgn0034126	FBtr0087157	170,81	1,82E-11	7,64E-15	2,59E-11	3,49	3,49
FBgn0264987	FBtr0335457	86,02	0,0000521	0,000000134	0,000074	3,21	3,21
FBgn0026197	FBtr0110844	85,74	0,0184	0,000203	0,0261	3,12	3,12
FBgn0036679	FBtr0273313	163,43	4,78E-08	6E-11	6,79E-08	3,03	3,03
FBgn0262166	FBtr0087246	59,48	0,0284	0,000392	0,0403	2,86	2,86
FBgn0024491	FBtr0347200	181,55	0,0235	0,000292	0,0334	2,84	2,84
FBgn0040531	FBtr0339331	44,12	0,029	0,000419	0,0413	2,62	2,62
FBgn0037901	FBtr0308211	169,75	0,000242	0,000000711	0,000344	2,53	2,53
FBgn0033809	FBtr0087765	371,08	3,56E-08	3,72E-11	5,06E-08	2,49	2,49
FBgn0053460	FBtr0100642	65,62	0,000719	0,00000281	0,00102	2,43	2,43
FBgn0053113	FBtr0301540	1252,73	0,00019	5,43E-07	0,00027	2,33	2,33
FBgn0033809	FBtr0333151	414,24	0,00000572	1,28E-08	0,00000813	2,23	2,23
FBgn0263402	FBtr0309181	42,52	0,00303	0,0000148	0,00431	2,23	2,23
FBgn0036585	FBtr0303379	176,88	0,000355	0,00000114	0,000504	2,18	2,18
FBgn0262478	FBtr0304816	33,34	0,011	0,0000852	0,0156	2,12	2,12
FBgn0036731	FBtr0302208	51,85	0,0151	0,000148	0,0214	2,12	2,12
FBgn0028494	FBtr0301202	1614,32	1,69E-08	1,65E-11	2,41E-08	2,11	2,11
AOX down-regulated							

Gene_ID	Transcript_ID	Total_reads	Q-value	P-value	FDR_step_up	Ratio	Fold_change

(daGal4>AOX_vs_daGal4)	(daGal4>AOX_vs_daGal4)	(daGal4>AOX_vs_daGal4)	(daGal4>AOX_vs_daGal4)	(daGal4>AOX_vs_daGal4)
FBgn0026189	FBtr0333895	158,7	0,0111	0,0000869	0,0158	0,5	-2,01
FBgn0032685	FBtr0343824	525,63	5,64E-08	7,87E-11	8,02E-08	0,49	-2,04
FBgn0036747	FBtr0075195	36,23	0,00677	0,0000454	0,00962	0,49	-2,05
FBgn0036279	FBtr0333839	167,21	0,0208	0,000248	0,0296	0,48	-2,1
FBgn0000308	FBtr0309212	69,42	0,00759	0,000054	0,0108	0,47	-2,12
FBgn0003495	FBtr0085146	148,47	0,0142	0,000134	0,0201	0,47	-2,12
FBgn0034010	FBtr0087390	63,06	0,000438	0,00000152	0,000623	0,47	-2,13
FBgn0002868	FBtr0334686	1325,99	0,000438	0,00000153	0,000623	0,47	-2,13
FBgn0034885	FBtr0072103	81,78	0,00581	0,000034	0,00825	0,47	-2,13
FBgn0264598	FBtr0333532	544,8	0,0258	0,000333	0,0366	0,47	-2,14
FBgn0028984	FBtr0083142	256,3	0,00318	0,0000161	0,00452	0,46	-2,18
FBgn0037936	FBtr0082437	35,6	0,0282	0,000386	0,0401	0,46	-2,18
FBgn0250835	FBtr0335171	176,28	0,000000151	2,63E-10	2,14E-07	0,45	-2,23
FBgn0261402	FBtr0302293	207,71	0,00000286	6,18E-09	0,00000406	0,45	-2,23
FBgn0262742	FBtr0112803	168,04	0,0344	0,000562	0,0489	0,45	-2,24
FBgn0040256	FBtr0113321	2666,54	0,0271	0,000364	0,0386	0,44	-2,26
FBgn0013276	FBtr0082482	1363,75	0,0023	0,0000109	0,00326	0,44	-2,29
FBgn0040582	FBtr0084180	2431,23	0,0131	0,000115	0,0186	0,44	-2,29
FBgn0013275	FBtr0082512	1167,15	0,00318	0,000016	0,00452	0,43	-2,3
FBgn0030970	FBtr0074653	97,57	0,0224	0,000272	0,0318	0,44	-2,3
FBgn0004646	FBtr0309875	516,49	0,033	0,000527	0,0468	0,43	-2,31
FBgn0267485	FBtr0346857	370,53	0,000124	0,000000337	0,000176	0,43	-2,32
FBgn0030318	FBtr0073519	129,13	0,0299	0,000456	0,0424	0,43	-2,33
FBgn0265194	FBtr0339082	81,82	0,0284	0,000395	0,0403	0,42	-2,37
FBgn0029092	FBtr0088549	380,28	0,0121	0,000102	0,0173	0,42	-2,38
FBgn0264449	FBtr0332611	39,41	0,0294	0,000438	0,0418	0,41	-2,41
FBgn0037415	FBtr0078598	130,85	0,000000132	2,02E-10	0,000000187	0,41	-2,44
FBgn0052251	FBtr0073286	52,26	0,013	0,000112	0,0185	0,4	-2,5
FBgn0262866	FBtr0077360	169,99	0,0176	0,00019	0,0249	0,39	-2,55
FBgn0032835	FBtr0081302	2611,83	0,000829	0,00000338	0,00118	0,38	-2,62
FBgn0036199	FBtr0076114	136,75	0,0288	0,00041	0,0409	0,37	-2,67
FBgn0022355	FBtr0074559	45822,77	0,0116	0,0000913	0,0164	0,37	-2,68
FBgn0037901	FBtr0082402	68,09	0,00436	0,0000237	0,00619	0,37	-2,7
FBgn0040360	FBtr0070200	43,71	0,00962	0,0000704	0,0137	0,37	-2,7
FBgn0016754	FBtr0308564	90,58	0,00517	2,92E-05	0,00735	0,37	-2,71
FBgn0032835	FBtr0346480	1819,21	0,0067	0,0000426	0,00952	0,36	-2,81
FBgn0002868	FBtr0082158	17075,94	0,00000193	4,04E-09	0,00000274	0,34	-2,94
FBgn0033402	FBtr0345024	122,63	0,0332	0,000534	0,0472	0,34	-2,94
FBgn0017550	FBtr0345140	99,63	0,0136	0,000127	0,0193	0,33	-3,03
FBgn0050101	FBtr0086963	163,56	3,36E-12	1,17E-15	4,77E-12	0,31	-3,27
FBgn0261239	FBtr0081480	74,42	0,0144	0,000138	0,0205	0,31	-3,28
FBgn0259734	FBtr0300001	79,43	0,0118	0,0000966	0,0168	0,29	-3,39
FBgn0033835	FBtr0087654	1399	0,0261	0,000344	0,037	0,29	-3,44
FBgn0053470	FBtr0303099	1477,46	0,0261	0,000344	0,037	0,29	-3,44
FBgn0034328	FBtr0086729	812,2	0,029	0,000417	0,0413	0,26	-3,79
FBgn0032685	FBtr0081069	123,11	0,0288	0,000409	0,0409	0,26	-3,84
FBgn0085422	FBtr0340590	71,18	0,0196	0,000226	0,0279	0,26	-3,9
FBgn0085443	FBtr0310291	58,21	0,0161	0,000164	0,0229	0,26	-3,91
FBgn0036015	FBtr0076421	14584,95	1,17E-10	5,7E-14	1,66E-10	0,24	-4,17
FBgn0030773	FBtr0074364	4873,48	8,94E-08	1,31E-10	1,27E-07	0,24	-4,19
FBgn0050033	FBtr0088117	588,59	1,13E-09	7,92E-13	1,61E-09	0,23	-4,28
FBgn0013279	FBtr0082638	188,02	4,49E-08	5,22E-11	6,38E-08	0,2	-4,95
FBgn0010385	FBtr0088432	91,27	0,00451	0,000025	0,0064	0,2	-5,11
FBgn0052523	FBtr0077183	461,14	0,00000057	1,11E-09	0,000000809	0,19	-5,2
FBgn0036016	FBtr0076380	61,73	3,05E-09	2,56E-12	4,34E-09	0,19	-5,38
FBgn0034490	FBtr0086320	59,27	0,0122	0,000103	0,0173	0,18	-5,56
FBgn0036022	FBtr0076387	8769,64	2,04E-10	1,28E-13	2,9E-10	0,11	-8,96
FBgn0039703	FBtr0085531	922,2	3,05E-09	2,54E-12	4,34E-09	0,1	-9,57
FBgn0040502	FBtr0085988	8452,06	1,59E-10	8,88E-14	2,26E-10	0,1	-10,03
FBgn0033067	FBtr0086005	837,08	5,07E-09	4,59E-12	7,2E-09	0,07	-14,09

To confirm that shortened survival under stress was a result of lower ROS levels and not secondary effects of AOX expression; we decreased the concentration of ROS by overexpressing antioxidants. We manipulated the levels of mitochondrial superoxide and H_2_O_2_ independently by overexpressing Sod2 and mtCAT, respectively. This strategy also allowed us to determine if a particular ROS had a leading role in stress signalling. Overexpression of mtCAT decreased mitochondrial H_2_O_2_ without altering superoxide levels (Fig. 2E, G). Like AOX expression, mtCAT dramatically shortened survival under severe and moderate heat stress (Fig. 3K and L). However, overexpression of Sod2 did not reduce lifespan, and in fact, a modest but significant extension was observed (Fig. 3M, N). It is worth noting that Sod2 decreases superoxide levels and in the process increases H_2_O_2_ (Figs. 2G and 1D). This data suggests that H_2_O_2_ is acting as the messenger ROS and is required for survival under stress conditions.

Our results demonstrate that interruption of ROS-RET signalling diminishes the response to long-term heat stress compromising the survival of flies under continuous heat stress.

### 3.4 ROS-RET is required for survival under hypoxic and hyperoxic conditions

**Fig. 4:**
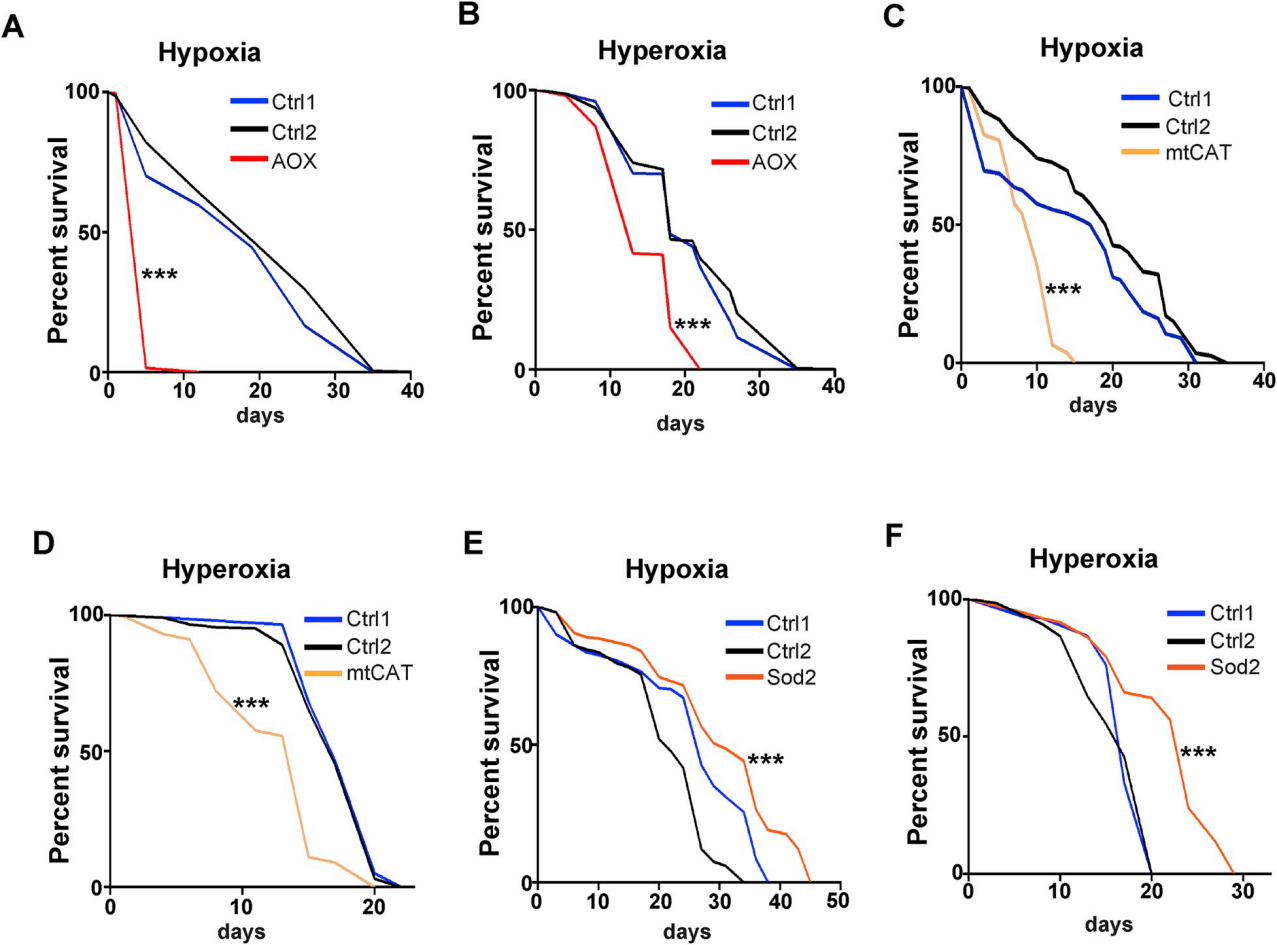
Survival under hypoxic and hyperoxic condition require ROS-RET activation. **A)** Survival of control (Ctrl1 = AOX>3 and Ctrl2 = 2>daGAL4) and AOX (AOX > daGAL4) flies cultured under hypoxia (5% O_2_). **B)** Survival of control (Ctrl1 = AOX>3 and Ctrl2 = 2>daGAL4) and AOX (AOX > daGAL4) flies cultured under hyperoxia (50% O_2_). **C)** Survival of control (Ctrl1 = mtCAT>3 and Ctrl2 = 2>daGAL4) and mtCAT (mtCAT > daGAL4) cultured under hypoxia (5% O_2_). **D)** Survival of control (Ctrl1 = mtCAT>3 and Ctrl2 = 2>daGAL4) and mtCAT flies (mtCAT > daGAL4) cultured under hyperoxia (50% O_2_). **E)** Survival of control (Ctrl1 = Sod2>3 and Ctrl2 = 2>daGAL4) and Sod2 flies (Sod2>daGAL4) cultured under hypoxia (5% O_2_). **F)** Survival of control (Ctrl1 = Sod2>3 and Ctrl2 = 2>daGAL4) and Sod2 flies (Sod2>daGAL4) cultured under hyperoxia (50% O_2_). Lifespan data were analysed using the log-rank Mantel Cox Test. *p < 0.05, **p < 0.01, ***p < 0.001.

Our transcriptomic data suggest that the general response to stress could be impaired upon disruption of ROS signalling. For example, several genes associated with “Response to hypoxia” (GO0001666) were downregulated in AOX flies (Fig. 3B). To test whether abolishing the ROS-RET signal could compromise the capacity of flies to respond to other types of stress, we either decreased (hypoxia) or increased (hyperoxia) the concentration of the oxygen. AOX expression severely shortened the lifespan of flies under hypoxia (Fig. 4A). Survival was also shortened by AOX expression under hyperoxia but to a lesser degree (Fig. 4B). As with AOX expressing flies, the survival of flies overexpressing mtCAT was severely shortened under hypoxia and more modestly shortened under hyperoxia (Fig. 4C and D). In line with survival under heat stress, overexpression of Sod2 extended survival under hyperoxia, and had a positive but modest effect in hypoxic conditions (Fig. 4E and F). We have previously shown that co-expression of mtCAT and NDI1 abolishes the lifespan extension conferred by NDI1 expression [13]. Indicating that elevated concentrations of mitochondrial H_2_O_2_ are necessary for the lifespan extension effects of NDI1. The work presented here further demonstrates that H_2_O_2_ is the ROS required for stress signalling and that it is upstream of adaptations coordinated at the transcriptomic level in response to stress. Within the mitochondrial ROS signalling pathway, we have demonstrated that Sod2 has a central role transforming superoxide into H_2_O_2_, which allows diffusion of the signal directly [31] or through peroxiredoxin-mediated signalling [32]. Importantly, our work explains why antioxidant therapies have failed to extend lifespan [2]. Although antioxidants can protect against oxidative damage (as Sod2 does under hyperoxia, Fig. 4F), suppression of ROS signalling mediated by H_2_O_2_ has negative consequences since it prevents full deployment of the adaptive stress response at the transcriptomic levels. Therefore, strategies which aim to reduce oxidative damage must also preserve ROS signalling if they are to be of benefit to health span and lifespan.

## 4 Conclusion

In recent years, several independent laboratories have provided evidence that ROS are not just by-products of metabolism which cause oxidative stress and underlie disease. ROS are essential messengers involved in deciding the developmental fate of cells and instrumental in tissue homeostasis [33]. It is these two opposing effects of ROS which make it extremely difficult to assert whether increased ROS levels are, the cause of a specific pathology, a consequence or conversely a redox distress signal intended to communicate and activate a cellular response. Here we show that exposure to heat stress leads to an increased ROS within the fly brain produced through RET via CI. Within a few hours of this initial increase, ROS levels return to normal suggesting that this is a specific signal in response to heat stress. In support of this hypothesis, the long-term disruption of the ROS-RET signal using expression of AOX diminishes the stress response, preventing the upregulation of many genes required to establish a pro-survival response resulting in a negative effect on the fly lifespan. Interestingly, mtCAT expression produces the same effect on longevity clarifying a major role of ROS and specifically H_2_O_2_ in stress adaptation and invites us to develop strategies where stimulation of ROS signalling could aid in the prevention, delay or reversal of ageing and age-related diseases. We propose the ROS-RET signal is likely required broadly for survival under stress. Also, our results strongly advise against the indiscriminate use of antioxidants that can lead to an abolition of ROS signalling and contribute to a reduction in the capacity to deal with stress. This is particularly relevant in the case of anti-ageing therapies since the main characteristic of ageing is the loss in the capacity to confront stress.

### Competing interests

The authors declare that they have no competing interests.

## Notes on author contributions

F.S. performed ROS, qPCR and lifespan experiments and prepared samples for transcriptomics. A. Sr. performed lifespan experiments. S.H.Y.L., R.V.S and L.M.M. performed transcriptomics analysis. R.S. performed qPCR and edited the final version of the manuscript. A. Sa. and F.S. wrote the manuscript and designed and supervised the project. F.S. assembled the figures. All authors contribute to the design and analysis of experiments, discussed the data and critically revised the manuscript.

## References

[1] Schieber M., Chandel N.S. (2014). ROS function in redox signaling and oxidative stress. Curr. Biol..

[2] Sanz A., Pamplona R., Barja G. (2006). Is the mitochondrial free radical theory of aging intact?. Antioxidants Redox Signal..

[3] Dell'agnello C., Leo S., Agostino A., Szabadkai G., Tiveron C., Zulian A., Prelle A., Roubertoux P., Rizzuto R., Zeviani M. (2007). Increased longevity and refractoriness to Ca(2+)-dependent neurodegeneration in Surf1 knockout mice. Hum. Mol. Genet..

[4] Owusu-Ansah E., Song W., Perrimon N. (2013). Muscle mitohormesis promotes longevity via systemic repression of insulin signaling. Cell.

[5] Schulz T.J., Zarse K., Voigt A., Urban N., Birringer M., Ristow M. (2007). Glucose restriction extends Caenorhabditis elegans life span by inducing mitochondrial respiration and increasing oxidative stress. Cell Metabol..

[6] Yang W., Hekimi S. (2010). A mitochondrial superoxide signal triggers increased longevity in Caenorhabditis elegans. PLoS Biol..

[7] Harman D. (2003). The free radical theory of aging. Antioxidants Redox Signal..

[8] Sena L.A., Chandel N.S. (2012). Physiological roles of mitochondrial reactive oxygen species. Mol. Cell..

[9] Hekimi S., Lapointe J., Wen Y. (2011). Taking a "good" look at free radicals in the aging process. Trends Cell Biol..

[10] Stefanatos R., Sanz A. (2018). The role of mitochondrial ROS in the aging brain. FEBS Lett..

[11] Murphy M.P. (2009). How mitochondria produce reactive oxygen species. Biochem. J..

[12] Schmeisser S., Priebe S., Groth M., Monajembashi S., Hemmerich P., Guthke R., Platzer M., Ristow M. (2013). Neuronal ROS signaling rather than AMPK/sirtuin-mediated energy sensing links dietary restriction to lifespan extension. Molecular metabolism.

[13] Scialo F., Sriram A., Fernandez-Ayala D., Gubina N., Lohmus M., Nelson G., Logan A., Cooper H.M., Navas P., Enriquez J.A. (2016). Mitochondrial ROS produced via reverse electron transport extend animal lifespan. Cell Metabol..

[14] Scialo F., Fernandez-Ayala D.J., Sanz A. (2017). Role of mitochondrial reverse electron transport in ROS signaling: potential roles in health and disease. Front. Physiol..

[15] Robb E.L., Hall A.R., Prime T.A., Eaton S., Szibor M., Viscomi C., James A.M., Murphy M.P. (2018). Control of mitochondrial superoxide production by reverse electron transport at complex I. J. Biol. Chem..

[16] Arias-Mayenco I., González-Rodríguez P., Torres-Torrelo H., Gao L., Fernández-Agüera M.C., Bonilla-Henao V., Ortega-Sáenz P., López-Barneo J. (2018). Acute O2 sensing: role of coenzyme QH2/Q ratio and mitochondrial ROS compartmentalization. Cell Metabol..

[17] Kempf A., Song S.M., Talbot C.B., Miesenbock G. (2019). A potassium channel beta-subunit couples mitochondrial electron transport to sleep. Nature.

[18] Mills E.L., Kelly B., Logan A., Costa A.S.H., Varma M., Bryant C.E., Tourlomousis P., Dabritz J.H.M., Gottlieb E., Latorre I. (2016). Succinate dehydrogenase supports metabolic repurposing of mitochondria to drive inflammatory macrophages. Cell.

[19] Dogan S.A., Cerutti R., Beninca C., Brea-Calvo G., Jacobs H.T., Zeviani M., Szibor M., Viscomi C. (2018 Nov 6). Perturbed redox signaling exacerbates a mitochondrial myopathy. Cell Metabol..

[20] Chouchani E.T., Pell V.R., Gaude E., Aksentijević D., Sundier S.Y., Robb E.L., Logan A., Nadtochiy S.M., Ord E.N.J., Smith A.C. (2014). Ischaemic accumulation of succinate controls reperfusion injury through mitochondrial ROS. Nature.

[21] McDonald A.E., Vanlerberghe G.C., Staples J.F. (2009). Alternative oxidase in animals: unique characteristics and taxonomic distribution. J. Exp. Biol..

[22] Hakkaart G.A., Dassa E.P., Jacobs H.T., Rustin P. (2006). Allotopic expression of a mitochondrial alternative oxidase confers cyanide resistance to human cell respiration. EMBO Rep..

[23] Cannino G., El-Khoury R., Pirinen M., Hutz B., Rustin P., Jacobs H.T., Dufour E. (2012). Glucose modulates respiratory complex I activity in response to acute mitochondrial dysfunction. J. Biol. Chem..

[24] Gubina N., Naudi A., Stefanatos R., Jove M., Scialo F., Fernandez-Ayala D.J., Rantapero T., Yurkevych I., Portero-Otin M., Nykter M. (2018). Essential physiological differences characterise short- and long-lived strains of Drosophila melanogaster. J Gerontol A Biol Sci Med Sci.

[25] Fernandez-Ayala D.J., Sanz A., Vartiainen S., Kemppainen K.K., Babusiak M., Mustalahti E., Costa R., Tuomela T., Zeviani M., Chung J. (2009). Expression of the Ciona intestinalis alternative oxidase (AOX) in Drosophila complements defects in mitochondrial oxidative phosphorylation. Cell Metabol..

[26] Huang da W., Sherman B.T., Lempicki R.A. (2009). Systematic and integrative analysis of large gene lists using DAVID bioinformatics resources. Nat. Protoc..

[27] Thompson K., Mai N., Oláhová M., Scialó F., Formosa L.E., Stroud D.A., Garrett M., Lax N.Z., Robertson F.M., Jou C. (2018). OXA1L mutations cause mitochondrial encephalopathy and a combined oxidative phosphorylation defect. EMBO Mol Med. Nov.

[28] Sanz A., Fernandez-Ayala D.J., Stefanatos R.K., Jacobs H.T. (2010). Mitochondrial ROS production correlates with, but does not directly regulate lifespan in Drosophila. Aging (Albany NY).

[29] Kang H.L., Benzer S., Min K.T. (2002). Life extension in Drosophila by feeding a drug. Proc. Natl. Acad. Sci. U. S. A..

[30] West A.P., Brodsky I.E., Rahner C., Woo D.K., Erdjument-Bromage H., Tempst P., Walsh M.C., Choi Y., Shadel G.S., Ghosh S. (2011). TLR signalling augments macrophage bactericidal activity through mitochondrial ROS. Nature.

[31] Lee S., Tak E., Lee J., Rashid M.A., Murphy M.P., Ha J., Kim S.S. (2011). Mitochondrial H2O2 generated from electron transport chain complex I stimulates muscle differentiation. Cell Res..

[32] Stocker S., Maurer M., Ruppert T., Dick T.P. (2017). A role for 2-Cys peroxiredoxins in facilitating cytosolic protein thiol oxidation. Nat. Chem. Biol..

[33] Reczek C.R., Chandel N.S. (2014). ROS-dependent signal transduction. Curr. Opin. Cell Biol..

